# Optimization and Characterization of TSG-Enriched *Polygonum multiflorum* Extract and Its Dual Mechanism Against Androgenetic Alopecia via 5α-Reductase Inhibition and Wnt/β-Catenin Activation

**DOI:** 10.3390/ijms27156648

**Published:** 2026-07-25

**Authors:** Te-Yang Huang, Min-Chieh Chang, Wen-Ta Su

**Affiliations:** 1 Department of Orthopedic Surgery, Mackay Memorial Hospital, Taipei 104217, Taiwan; haungt33@gmail.com; 2 Department of Chemical Engineering and Biotechnology, National Taipei University of Technology, Taipei 106344, Taiwan; cf1811@ntut.edu.tw

**Keywords:** *Polygonum multiflorum* Thunb., 5α-reductase, TSG, hair growth, Wnt/β-catenin pathway

## Abstract

This study aimed to optimize the extraction and purification of TSG-enriched *Polygonum multiflorum* extract and evaluate its therapeutic potential against androgenetic alopecia. *P. multiflorum* Thunb. has long been used in traditional Chinese medicine to promote hair growth and preserve hair pigmentation. Microwave-assisted extraction (MAE) was optimized to obtain a 2,3,5,4′-tetrahydroxystilbene-2-O-β-D-glucoside (TSG)-enriched *P. multiflorum* extract (PME). The crude extract was further purified by medium-pressure liquid chromatography and semipreparative high-performance liquid chromatography. Phytochemical profiling by HPLC demonstrated a 3.75-fold increase in TSG content, from 22,770.0 ± 815 ppm to 85,387.5 ± 192 ppm, and the identity of the enriched TSG was confirmed by LC–MS and ^1^H NMR spectroscopy. PME exhibited potent dose-dependent inhibition of 5α-reductase activity, reaching 92.7% inhibition at 1 μg/mL, comparable to that of finasteride. In a testosterone (TES)-induced androgenetic alopecia (AGA) mouse model, PME markedly accelerated hair regrowth and increased both the number and size of hair follicles. Mechanistically, PME suppressed the conversion of TES to dihydrotestosterone (DHT) through 5α-reductase inhibition and promoted GSK3β inactivation, β-catenin stabilization, and nuclear translocation, indicating activation of the Wnt/β-catenin signaling pathway. Consequently, the expression of hair growth-related proteins, including Ki67 (1.98-fold), epidermal growth factor (EGF, 1.54-fold), insulin-like growth factor-1 (IGF-1, 1.21-fold), and vascular endothelial growth factor (VEGF, 1.34-fold), was significantly upregulated. These findings demonstrate, for the first time, that TSG-enriched PME obtained through optimized MAE and chromatographic purification promotes hair regeneration through a dual mechanism involving 5α-reductase inhibition and Wnt/β-catenin activation, highlighting its potential as a natural therapeutic candidate for androgenetic alopecia.

## 1. Introduction

Androgenetic alopecia (AGA) is a hereditary disorder and one of the most prevalent causes of hair loss worldwide. Its characteristic symptoms include progressive miniaturization of hair follicles and hair thinning, which markedly shorten the hair follicle growth cycle. In severe cases, hair loss may advance to complete baldness [1]. These symptoms often appear or worsen during or after puberty, and the prevalence increases with age—approximately 50% of men over 50 years experience AGA [2]. Although AGA is not directly associated with adverse physiological health outcomes, it exerts a profound negative impact on quality of life, self-image, and psychological well-being [3].

Androgens are essential for bone and muscle development, sexual differentiation, and reproductive function, and also play a critical role in regulating hair growth. The primary androgens involved are testosterone (TES) and its metabolites, dihydrotestosterone (DHT) and estradiol [4,5]. DHT exhibits a markedly higher affinity for androgen receptors compared to TES, leading to stronger biological effects. Elevated DHT levels have been implicated in several androgen-related disorders, including benign prostatic hyperplasia, prostate cancer, and AGA [6].

Although multiple signaling pathways, such as Wnt/β-catenin, TGF-β, and PI3K/Akt, contribute to AGA pathogenesis, the underlying mechanism remains incompletely understood. Elevated levels of DHT and 5α-reductase have been consistently reported in AGA patients [7]. DHT is synthesized from TES via the enzyme 5α-reductase and competes with TES for binding to androgen receptors on dermal papilla cells, with a binding affinity approximately five times higher than that of TES [8]. Excessive 5α-reductase activity promotes persistent DHT binding, leading to dysregulation of the hair follicle cycle, follicular atrophy, and apoptosis, ultimately resulting in AGA. Currently, finasteride and minoxidil are the two most commonly prescribed FDA-approved drugs for AGA treatment. Finasteride acts as a selective 5α-reductase inhibitor that effectively decreases DHT production and is clinically used for both benign prostatic hyperplasia and AGA [8]. Minoxidil, a potassium channel opener, enhances blood flow around hair follicles by promoting vasodilation [9]. Treatment with 2% or 5% minoxidil has been shown to upregulate the Wnt/β-catenin signaling pathway and vascular endothelial growth factor (VEGF) expression, thereby promoting hair follicle proliferation and prolonging the anagen phase [10]. However, these drugs may cause side effects, and their efficacy varies among individuals, necessitating the search for safer alternatives. Medicinal plants represent a rich source of bioactive compounds, including polyphenols, flavonoids, stilbenes, and other phytochemicals, which exhibit diverse pharmacological activities such as antioxidant, anti-inflammatory, antimicrobial, and regenerative effects. These plant-derived compounds have long been central to traditional medicine systems and continue to play a crucial role in modern drug discovery and development. In recent years, increasing attention has been directed toward natural bioactive compounds as promising alternatives to synthetic drugs, owing to their multi-target mechanisms of action, lower toxicity, and improved biocompatibility. Emerging studies have highlighted their potential in preventing and managing chronic diseases, including metabolic disorders, inflammation, and dermatological conditions such as hair loss. Furthermore, advances in phytochemical research and extraction technologies have facilitated the identification, isolation, and functional characterization of these compounds, enhancing their applicability in nutraceuticals and therapeutic formulations. The recent literature also emphasizes the role of plant bioactives in modulating key molecular pathways related to oxidative stress, immune response, and tissue regeneration, further supporting their therapeutic relevance [11,12,13]. Therefore, the exploration of bioactive compounds from medicinal plants, together with the optimization of sustainable extraction methods, remains a critical area for the development of effective and safe therapeutic strategies [14,15].

*Polygonum multiflorum* Thunb. (PM), belonging to the Polygonaceae family, has been widely used in traditional Chinese medicine for centuries and is distributed throughout East Asia. The tuberous root of PM, known as He Shou Wu, is traditionally used to nourish the liver and kidneys, supplement the diet, and prevent or treat premature graying and hair loss and delay aging. PM exhibits diverse pharmacological activities, including anti-atherosclerotic [16], antioxidant [17], anti-inflammatory [18], and anti-apoptotic properties, anticancer, hypolipidemic, and vasodilatory effects, and has been reported to prevent bone loss [19,20,21,22]. Its major bioactive constituents include emodin, chrysophanol, and 2,3,5,4′-tetrahydroxystilbene-2-O-β-D-glucoside (TSG), along with minor components such as anthraquinones, flavonoids, polyphenols, and lecithin. Previous studies have demonstrated that PM promotes hair growth by activating the Sonic Hedgehog (Shh) and β-catenin signaling pathways to stimulate hair follicles to transition from the telogen phase to the anagen phase and darkens hair by stimulating melanin synthesis through the MC1R/MITF/tyrosinase pathway [23]. However, PM has certain hepatotoxicity, and its safety in clinical use has always been a major concern [24,25].

Extraction of bioactive phytochemicals from medicinal plants can be achieved using conventional techniques, such as maceration, reflux extraction, and Soxhlet extraction, as well as modern green extraction technologies, including ultrasound-assisted extraction (UAE), microwave-assisted extraction (MAE), supercritical fluid extraction (SFE), and pressurized liquid extraction (PLE). Conventional extraction methods are generally simple and widely used but often require prolonged extraction times, large volumes of organic solvents, and high energy consumption, which may reduce extraction efficiency and increase the degradation of thermolabile compounds. In contrast, modern extraction techniques have been developed to improve extraction efficiency while reducing solvent usage and processing time. Among these techniques, MAE utilizes microwave energy to rapidly heat the extraction solvent and plant matrix through ionic conduction and dipole rotation, resulting in enhanced mass transfer and efficient release of intracellular phytochemicals [26]. Compared with conventional extraction methods, MAE offers several advantages, including shorter extraction time, lower solvent consumption, higher extraction yield, and improved energy efficiency. Moreover, previous studies have demonstrated that MAE is particularly suitable for extracting phenolic compounds and stilbene glucosides from medicinal plants [27]. Therefore, MAE was selected in this study as a green and efficient extraction approach for enriching TSG from *P*. *multiflorum*.

The novelty of this study lies in integrating optimized MAE, chromatographic purification, and structural characterization with comprehensive biological and mechanistic evaluations of Polygonum multiflorum extract. To the best of our knowledge, this is the first study to demonstrate that TSG-enriched *Polygonum multiflorum* extract (PME), obtained through optimized extraction and purification procedures, simultaneously suppresses androgen signaling via 5α-reductase inhibition and promotes hair follicle regeneration through activation of the Wnt/β-catenin signaling pathway. Accordingly, the present study aimed to optimize the extraction and purification of TSG-enriched PME, characterize the purified extract by LC–MS and ^1^H NMR spectroscopy, and evaluate its therapeutic potential against AGA. The bioactivity of PME was first assessed by determining its inhibitory effect on 5α-reductase activity. Subsequently, the hair growth-promoting efficacy of PME was investigated in TES-induced C57BL/6 mice, and the expression of proteins associated with the Wnt/β-catenin signaling pathway and hair growth was analyzed by Western blotting to elucidate the underlying molecular mechanisms.

## 2. Results

### 2.1. Optimization of MAE Parameters for P. multiflorum Thunb.

**Figure 1:**
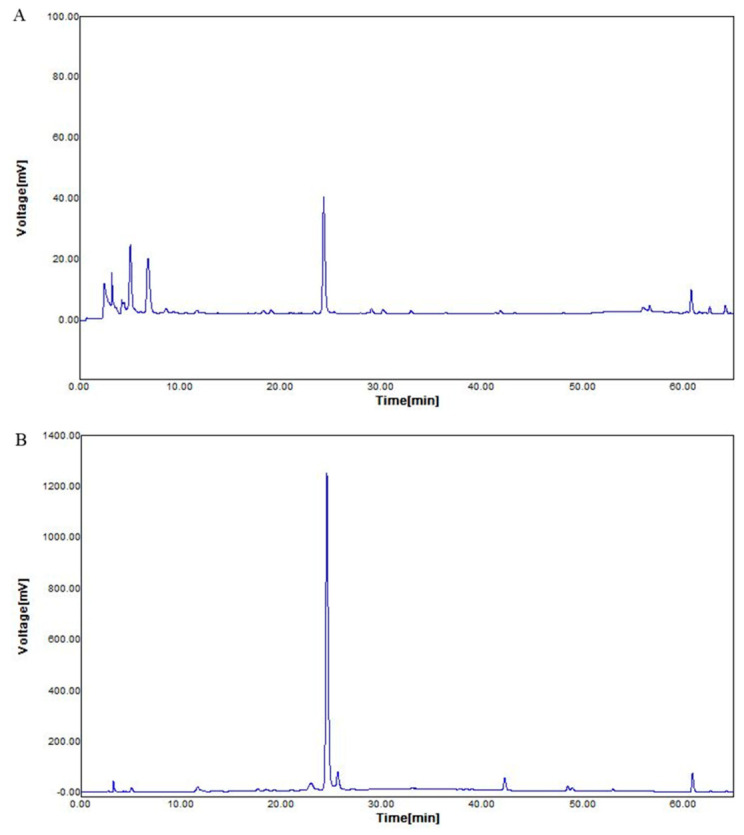
HPLC chromatograms indicate that raw *P. multiflorum* (**A**) contains higher levels of active ingredients than processed *P. multiflorum* (**B**).

The contents of active compounds in raw and processed PM were first compared. The experimental results showed that the yield of TSG from raw PM was markedly higher than that from the processed PM (Figure 1). Therefore, raw PM was selected for subsequent experiments.

Using 2 g portions of the ground sample, the MAE parameters-including ethanol concentration, extraction time, temperature, solvent volume, and microwave power-were optimized for maximum TSG yield. The baseline extraction conditions were set at 20 min, 70 °C, a solvent-to-solid ratio of 40:1 (*v*/*w*), and 600 W microwave power. The ethanol concentration was subsequently varied to identify the optimal extraction condition.

**Figure 2:**
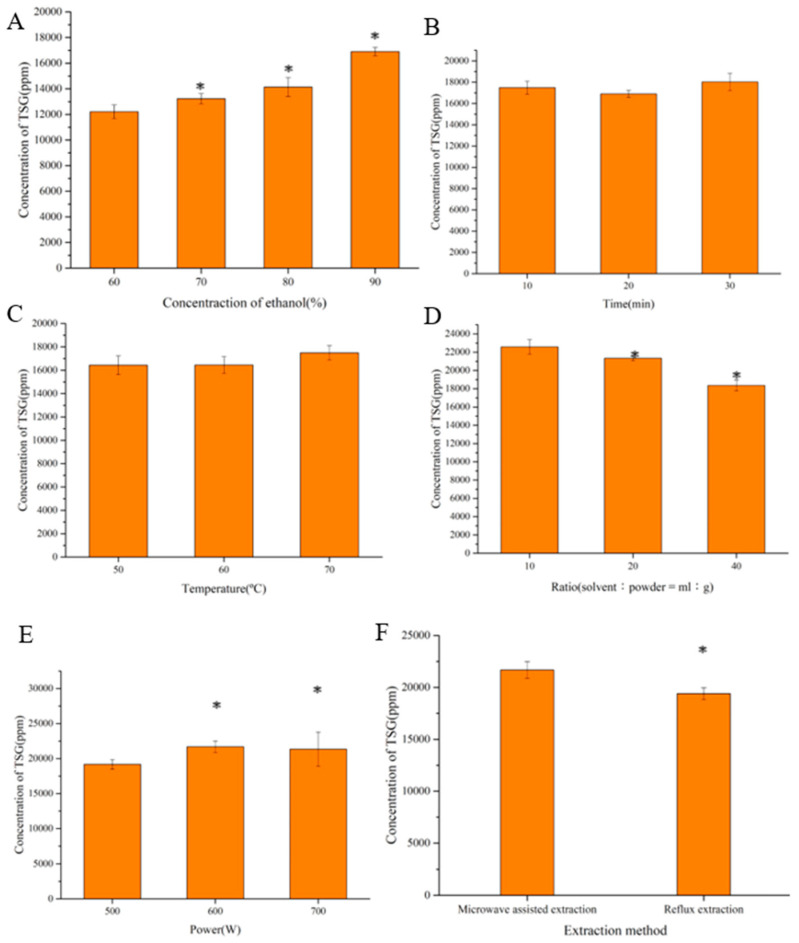
Optimization of MAE for TSG extraction. (**A**) Effect of ethanol concentration (temperature = 70 °C; extraction time = 20 min; microwave power = 600 W; solvent-to-solid ratio = 40 mL/g). (**B**) Effect of extraction temperature (ethanol = 90%; extraction time = 20 min; microwave power = 600 W; solvent-to-solid ratio = 40 mL/g). (**C**) Effect of extraction time (ethanol = 90%; temperature = 70 °C; microwave power = 600 W; solvent-to-solid ratio = 40 mL/g). (**D**) Effect of solvent-to-solid ratio (ethanol = 90%; temperature = 70 °C; extraction time = 20 min; microwave power = 600 W). (**E**) Effect of solvent-to-solid ratio (ethanol = 90%; temperature = 70 °C; extraction time = 20 min; solvent-to-solid ratio = 10 mL/g). (**F**) Comparative optimization of various extraction methods. The optimal conditions are: Reflux extraction: 90% ethanol, 40 min, solvent/solute = 10 (*v*/*w*), and Microwave extraction: 90% ethanol, 10 min, 70 °C, solvent/solute = 10 (*v*/*w*), 600 W. Data are presented as mean ± standard deviation (*n* = 3). * *p* < 0.05 compared with the first operating parameter.

Figure 2A–E illustrate the effects of different MAE parameters on the TSG yield of crude MAE extract solution. The optimal conditions were determined to be 90% ethanol, 10 min, 70 °C, 10 mL solvent per 2 g sample, and 600 W. Under these parameters, the TSG yield reached 22,770 ± 815 ppm. To further assess the advantages of MAE, a conventional reflux extraction was also performed, as shown in Figure 2F; the TSG yield from MAE was approximately 1.2-fold higher than that from reflux extraction, confirming that MAE was more efficient. Therefore, MAE was employed in all subsequent experiments.

### 2.2. Purification of PME and Compound Identification

**Figure 3:**
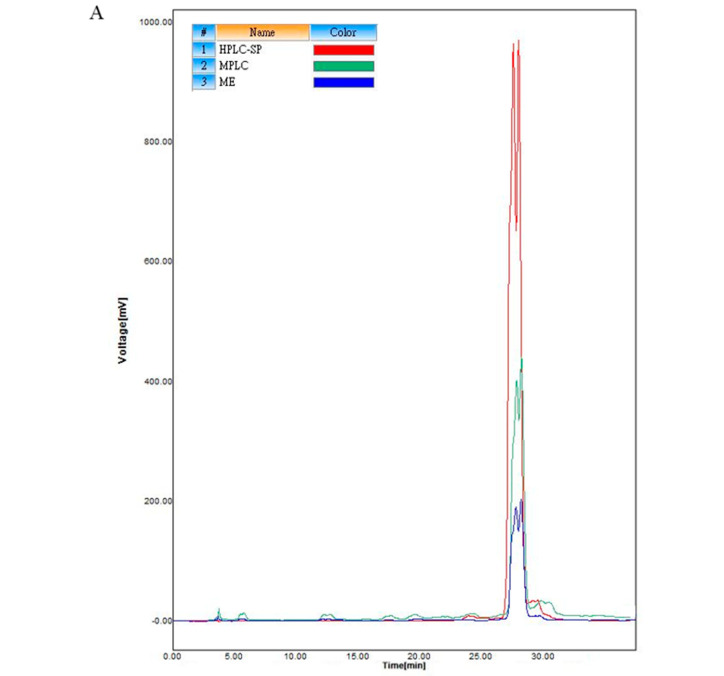

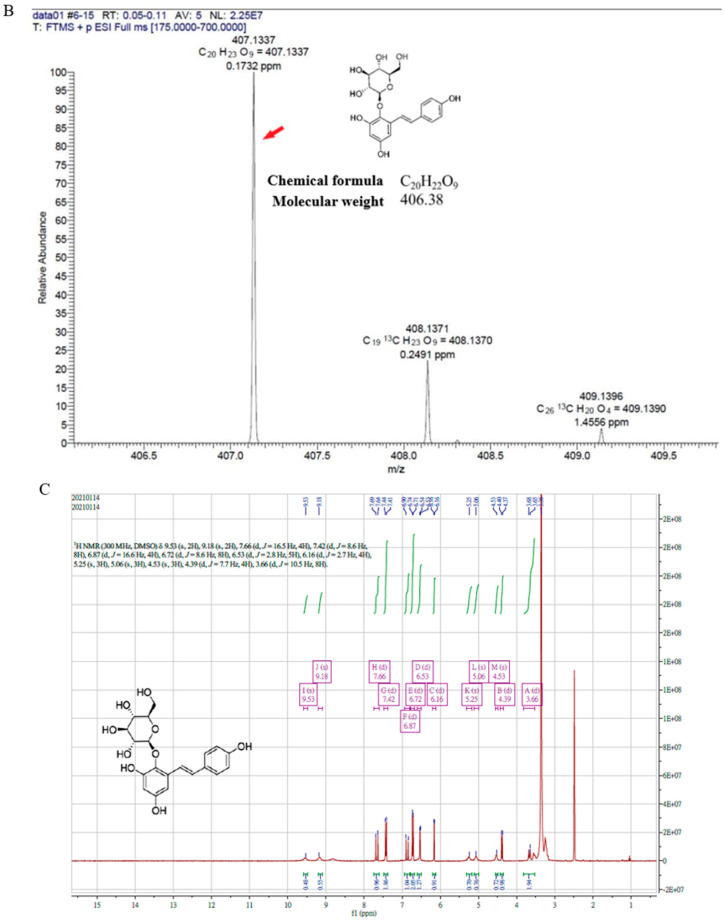
Chemical profiling of the extract following MAE, MPLC, and semipreparative HPLC purification (**A**); LC–MS identification profile (**B**); and ^1^H NMR identification profile (**C**).

The solution of crude MAE extract obtained via MAE was further purified using MPLC, followed by semipreparative HPLC, and the chromatographic purification procedure was designed to isolate TSG as the target compound. Minor constituents present in the crude extract were discarded during MPLC and semi-preparative HPLC purification and were therefore outside the scope of the present study. Structural identification of purified TSG by LC–MS and ^1^H NMR. The TSG content of the HPLC-purified solution significantly increased after each purification step. Figure 3A compares the extracts obtained from crude MAE extract, MPLC, and HPLC-purified PME. The TSG concentration increased to 85,387.5 ± 192 ppm in HPLC-purified solution-approximately 3.75-fold higher than that of the crude MAE extract solution, indicating a marked enhancement in purity and yield.

The chemical composition of the HPLC-purified PME was identified using LC–MS (Figure 3B) and ^1^H NMR spectroscopy (Figure 3C). LC–MS analysis revealed a molecular ion peak corresponding to C_20_H_22_O_9_, suggesting a stilbene glucoside derivative (molecular weight = 406.38), and the two peaks on the right are C_13_ isotope peaks. The ^1^H NMR spectrum (300 MHz, DMSO-d_6_) displayed two characteristic trans-olefinic protons at δ 7.66 and 6.87 (J ≈ 16 Hz), confirming the stilbene structure. The aromatic protons of the para-substituted B-ring appeared at δ 7.42 (H-2′,6′) and 6.72 (H-3′,5′). The A-ring exhibited meta-coupled signals at δ 6.53 (H-6) and 6.16 (H-4). The anomeric proton at δ ~5.0 (d, J ≈ 7.5 Hz) confirmed the β-D-glucoside linkage. Carbon Numbering: A-ring: C1–C6, B-ring: C1′–C6′, Glucose: C1″–C6″, confirming the presence of TSG.

### 2.3. Inhibitory Effect of PME on 5α-Reductase Activity

**Figure 4:**
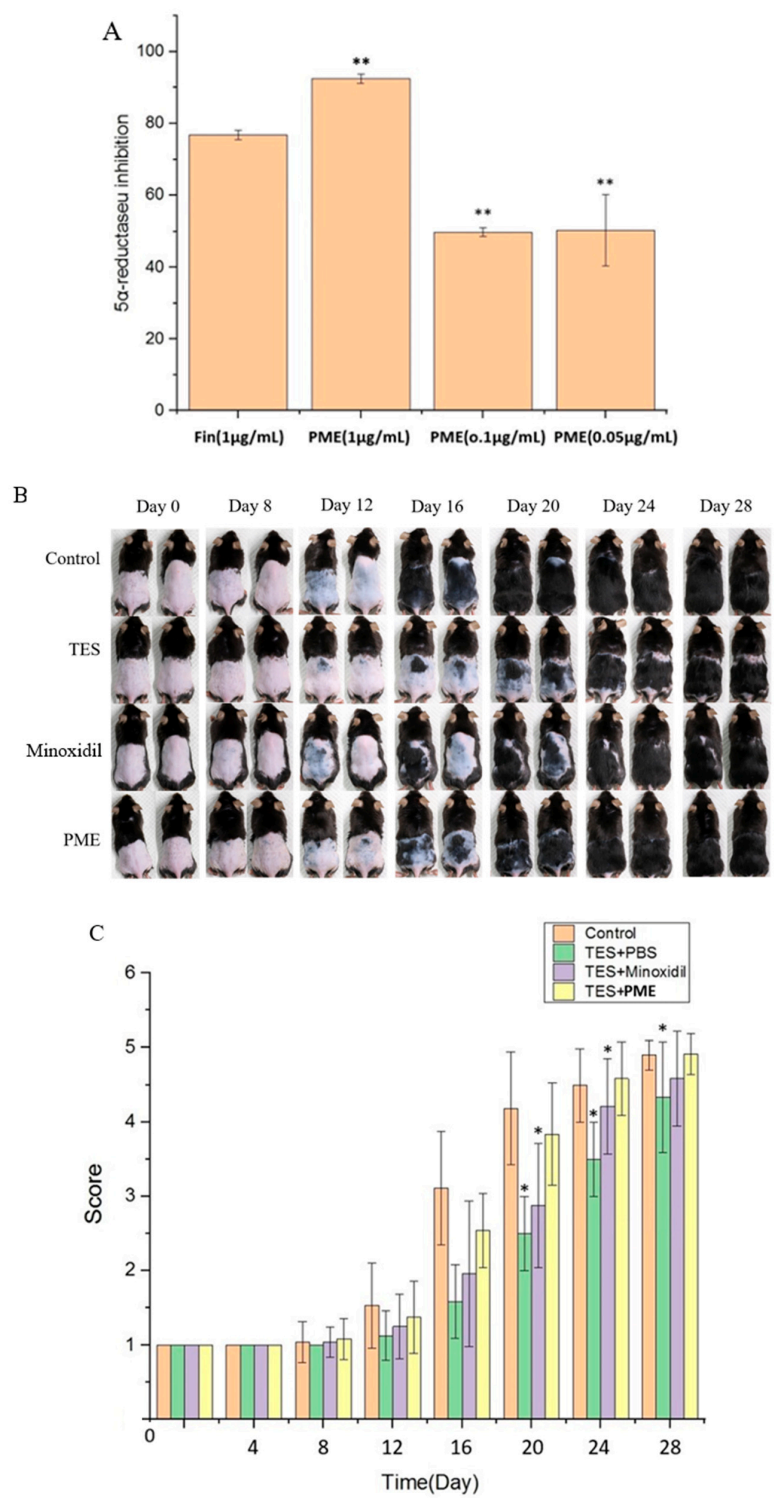
Inhibitory effects of finasteride and PME on 5α-reductase activity (**A**). Data are presented as mean ± standard deviation (*n* = 3). ** *p* < 0.01 compared with finasteride. Hair-regeneration effects of PME and minoxidil in C57BL/6 mice with TES-induced androgenetic alopecia (**B**). Hair growth efficacy was scored as 0, 1, 2, 3, 4, and 5, corresponding to 0%, 0–20%, 20–40%, 40–60%, 60–80%, and 80–100% hair coverage, respectively (**C**). * *p* < 0.05 compared with the control group.

PME inhibited 5α-reductase activity in a dose-dependent manner (Figure 4A). At a concentration of 1 µg/mL, PME achieved 92.7% inhibition of enzyme activity (n = 10), with an estimated IC_50_ of approximately 0.1 µg/mL. Notably, this inhibitory effect exceeded that of finasteride at the same concentration (77.2%), which was used as a selective 5α-reductase inhibitor and positive control. Based on these results, a PME concentration of 1 µg/mL was selected for subsequent in vivo experiments.

### 2.4. Effect of PME on Hair Growth in Mice

The dorsal hair of C57BL/6 mice exhibits a synchronized hair cycle, in which skin pigmentation serves as an indicator of phase transition: bright pink in the telogen phase and gray to black in the anagen phase. Initially, there was no significant change in skin color among all mice. On day 8, the Minoxidil and PME groups showed some gray deposits, and significant changes appeared after day 12. As shown in Figure 4B, the control group displayed gray skin by day 12 after depilation, visible short hair shafts by day 16, and complete coverage by day 28. In contrast, the TES-treated group exhibited delayed pigmentation and approximately 80–90% coverage at day 28, indicating suppressed hair regeneration. Topical application of PME effectively restored hair regrowth in TES-treated mice. Similar to the control group, PME-treated mice showed early pigmentation and full hair coverage by day 28. These results suggest that PME inhibits 5α-reductase-mediated conversion of TES to DHT, thereby restoring normal hair growth.

Hair growth in the minoxidil group was comparable to that in the control and PME groups. Although minoxidil promotes follicular growth, it does not inhibit 5α-reductase [28]; thus, its efficacy is limited under conditions of elevated TES. Quantitative assessment using the hair growth scoring system [29] showed that PME completely reversed the TES-induced suppression, yielding a score similar to the control and significantly higher than the TES group (Figure 4C).

**Figure 5:**
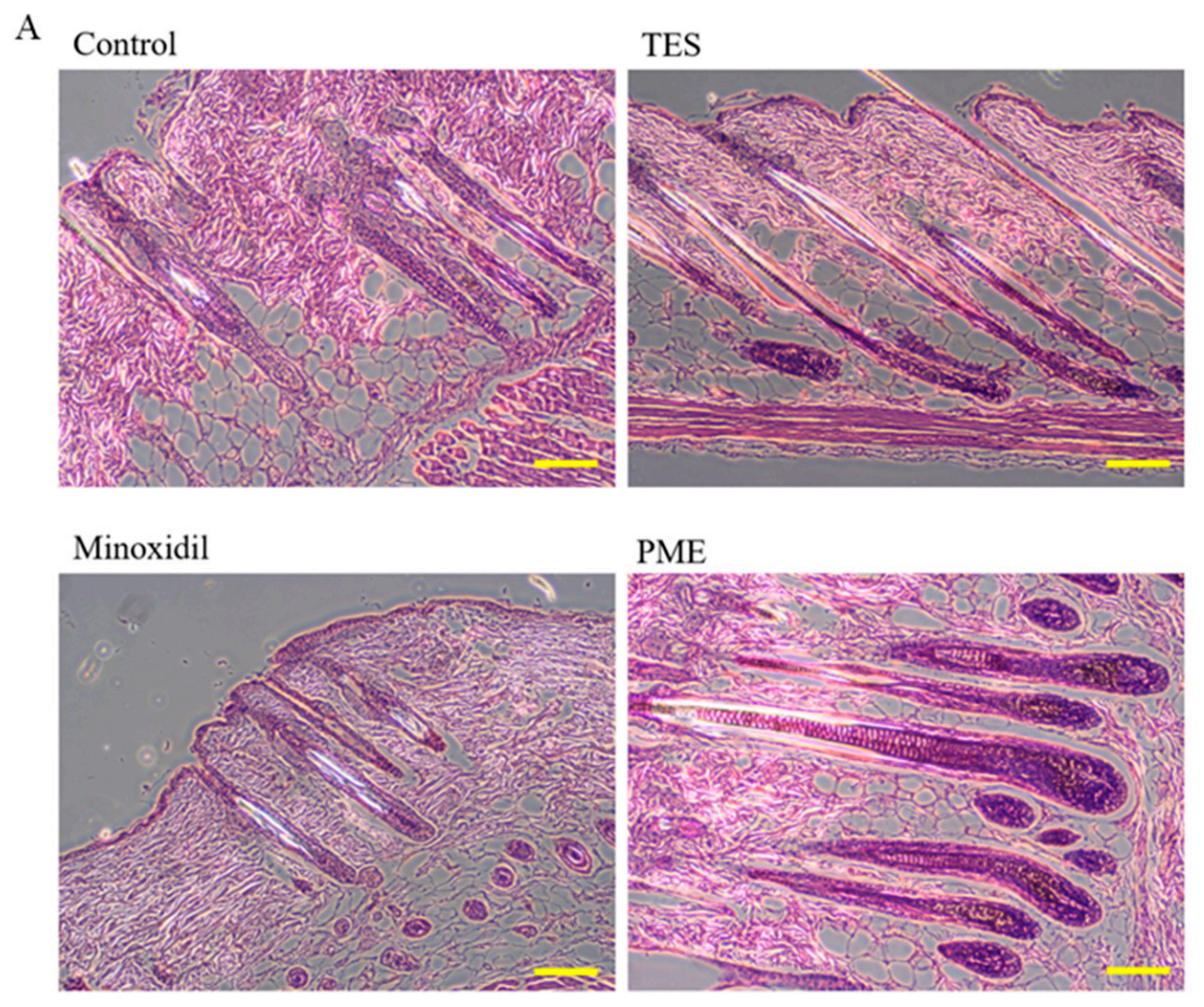

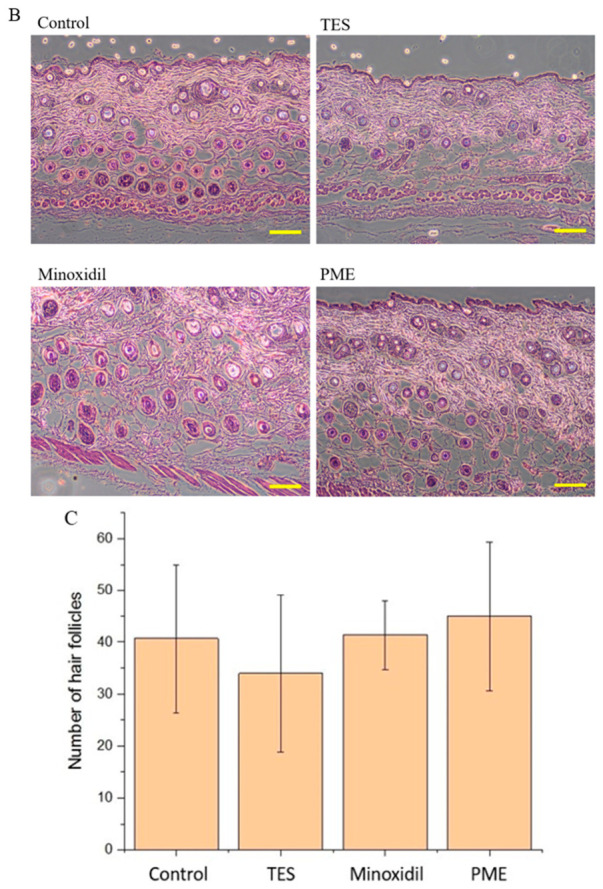
Hematoxylin–eosin staining showing that treatment with PME increased hair follicle size and number on day 28. Longitudinal sections (**A**) and cross-sectional views (**B**), scale bar = 100 μm. Quantification of hair follicles was conducted using three randomly selected microscopic fields per tissue sample (**C**), and data are presented as mean ± SD (*n* = 3). No statistically significant difference was observed compared with the control.

Histological analysis further confirmed these findings. By the end of the experiment, all groups displayed follicles in the mature anagen phase. Longitudinal and cross-sectional analyses revealed that hair follicles in the PME, control, and minoxidil groups were round and intact (Figure 5A,B), whereas follicles in the TES group appeared shrunken and atrophic, approximately 75–80% the size of controls, consistent with androgenetic alopecia. Quantitative analysis (Figure 5C) showed that the TES group had fewer follicles, while PME treatment restored follicle density to levels comparable to or exceeding those of the control and minoxidil groups. These findings confirm that PME enriched with TSG suppresses DHT formation and promotes follicular regeneration.

### 2.5. Effect of PME on Growth Factor Expression

**Figure 6:**
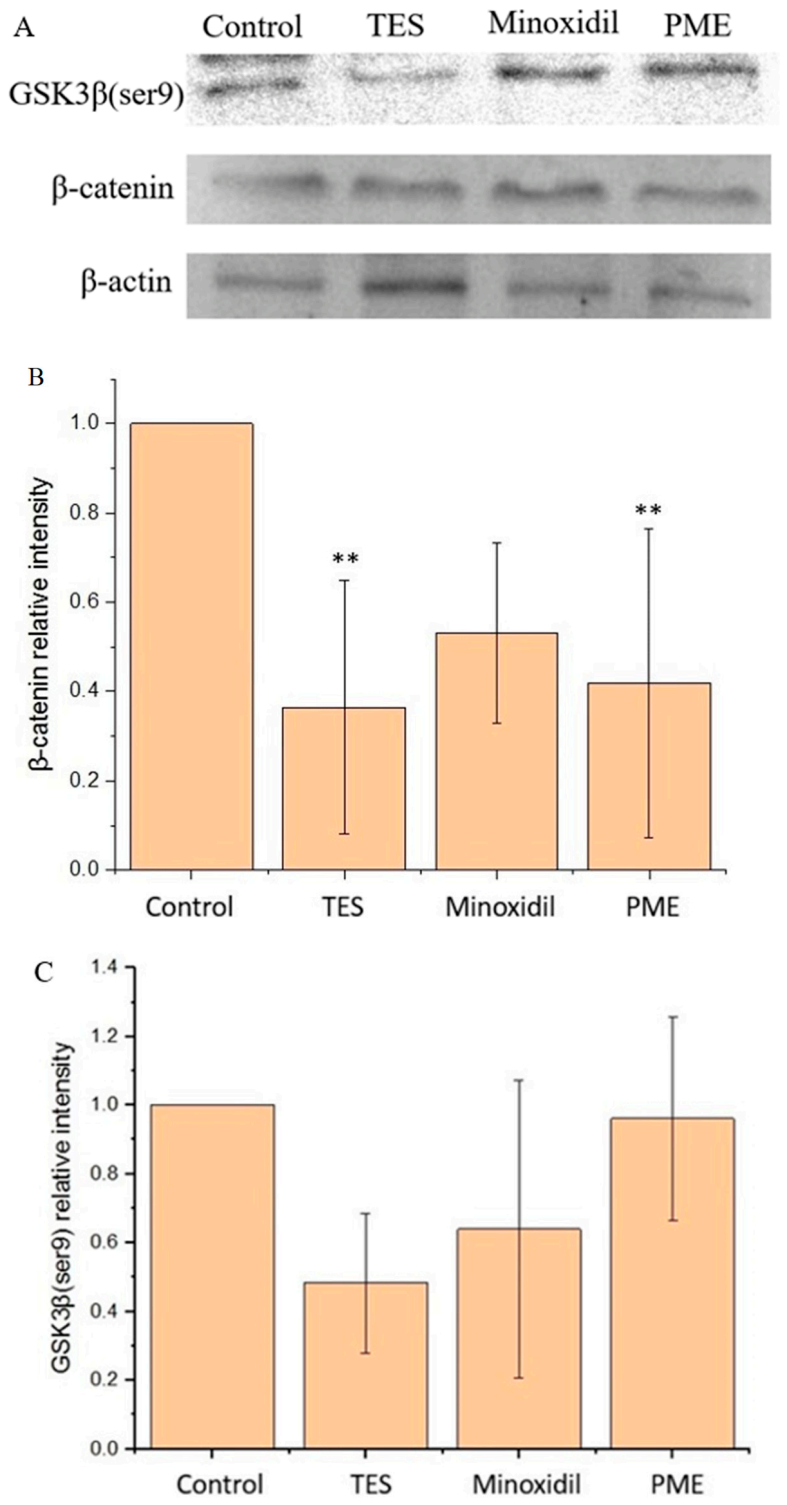
Western blot analysis of β-catenin and GSK3β (Ser9) expression in skin tissue from C57BL/6 mice with TES-induced androgenetic alopecia treated with PME or minoxidil (**A**). Band intensities of β-catenin (**B**) and GSK3β (Ser9) (**C**) were quantified by densitometry and normalized to β-actin. Data are presented as mean ± SD (*n* = 3). ** *p* < 0.01 compared with the control group.

The Wnt/β-catenin signaling pathway plays a key role in hair follicle activation and growth. As shown in Figure 6, the protein expression levels of β-catenin (Figure 6B) and phosphorylated GSK3β at Ser9 (Figure 6C) were significantly decreased in the TES group. However, PME treatment markedly restored these levels, with β-catenin reaching 46.0% and phosphorylated GSK3β (Ser9) reaching 98.9% of the control. In contrast, the minoxidil group showed lower recovery levels (14.9% and 32.4%, respectively). These results suggest that PME enhances GSK3β phosphorylation, thereby stabilizing β-catenin and activating Wnt/β-catenin signaling.

**Figure 7:**
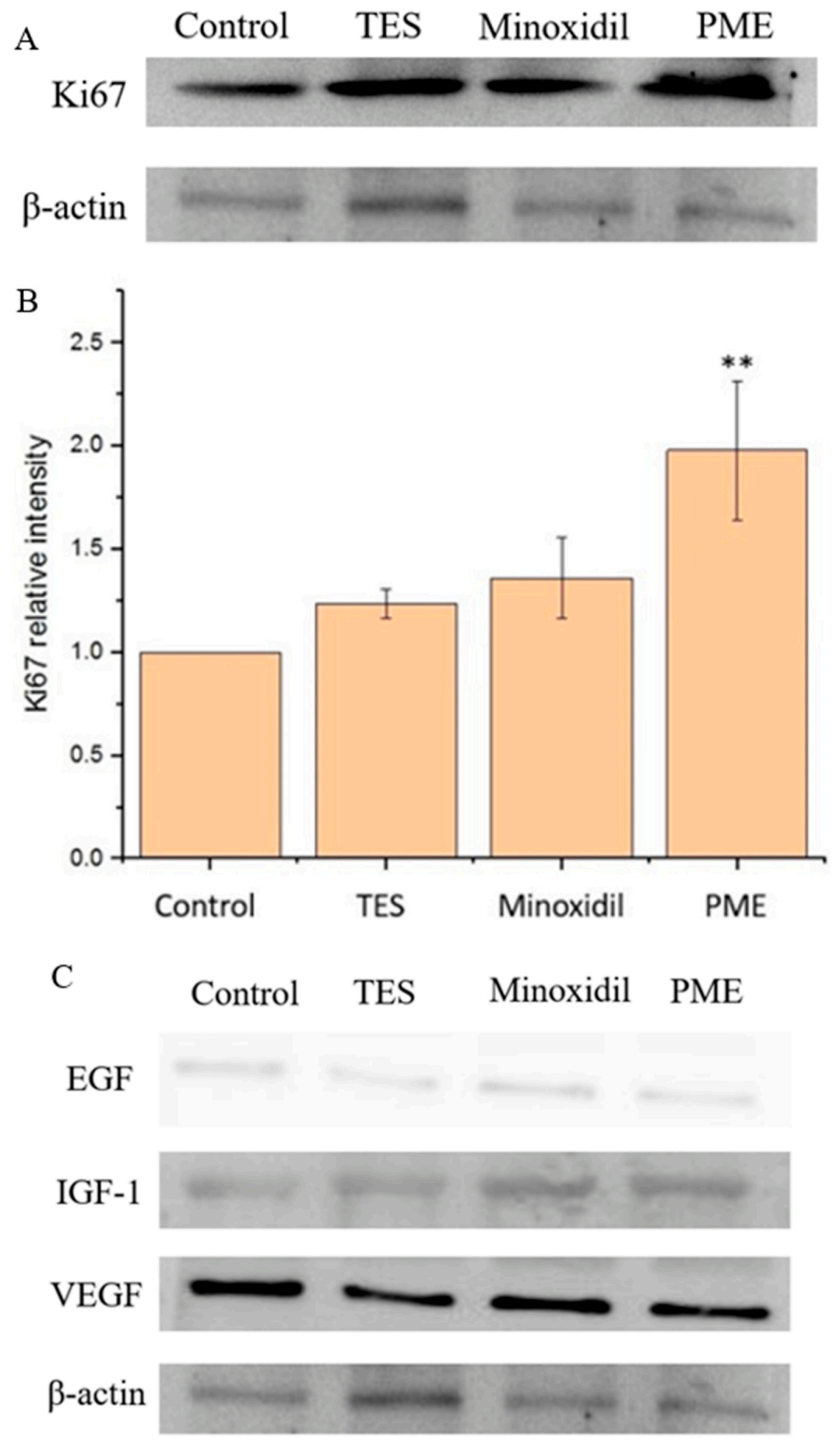

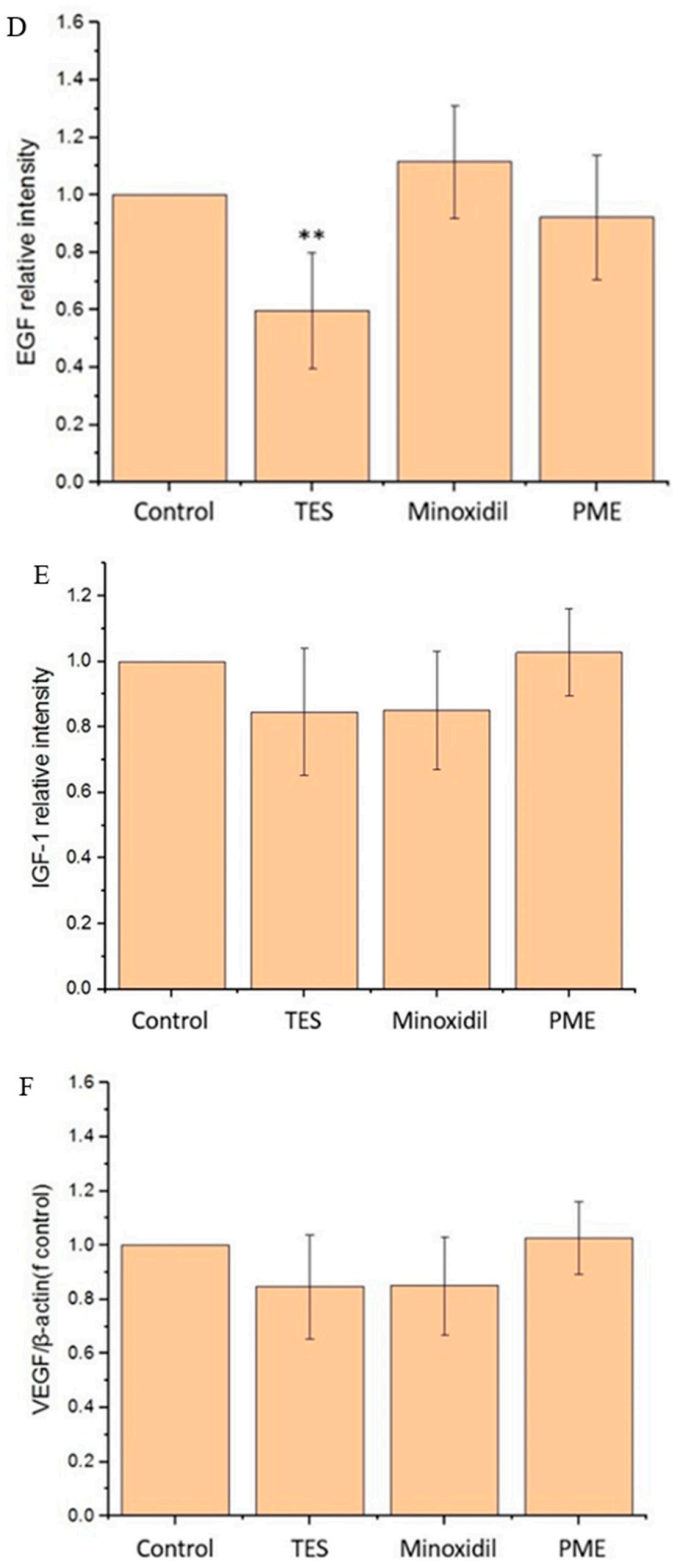
Western blot analysis of Ki67 (**A**), EGF, IGF-1, and VEGF (**C**) expression in skin tissue from C57BL/6 mice. Band intensities of Ki67 (**B**), EGF (**D**), IGF-1 (**E**), and VEGF (**F**) were quantified by densitometry and normalized to β-actin. Data are presented as mean ± SD (*n* = 3). ** *p* < 0.01 compared with the control group.

Ki67, a nuclear proliferation marker expressed during the active phases of the cell cycle (G1–M), was most abundantly expressed in the PME group after 28 days of treatment. The number of Ki67-positive follicular cells was 1.98-fold higher in the PME group than in the control (Figure 7A,B), indicating enhanced follicular cell proliferation.

Growth factors are important parameters in the hair follicle growth cycle and hair growth (Figure 7C). TES treatment reduced EGF levels, while minoxidil and PME treatments restored them by 1.86-fold and 1.54-fold, respectively (Figure 7D). PME also upregulated IGF-1 by 1.21-fold, whereas minoxidil had no significant effect (Figure 7E). Both PME and minoxidil markedly increased VEGF expression by 1.52-fold and 1.34-fold, respectively, promoting perifollicular angiogenesis and nutrient delivery (Figure 7F).

Collectively, these results demonstrate that PME promotes hair growth by modulating 5α-reductase activity, activating the Wnt/β-catenin signaling pathway, and upregulating growth factors associated with follicular proliferation and vascularization.

## 3. Discussion

Chinese herbal medicines and plant-derived extracts are widely applied in food, traditional medicine, and cosmetic formulations. These natural products, an important source of therapeutic agents, demonstrate superior efficacy, anti-inflammatory and immunomodulatory effects, as well as higher bioactivity and a lower incidence of adverse effects compared with conventional allopathic drugs, and can be considered as potential sources for alleviating and treating AGA [11]. Optimizing extraction parameters is crucial to maximizing yields of bioactive compounds, improving energy efficiency, and minimizing solvent consumption. TSG is an effective component in PM that promotes hair follicle growth [23,30]. Therefore, microwave extraction is the optimal method for extraction, followed by enrichment to extract the TSG concentration.

In this study, the concentration of TSG in the crude MAE extract obtained under optimized MAE conditions (90% ethanol, 10 min, 70 °C, 10 mL solvent per 2 g sample, and 600 W) reached 22,770 ± 815 ppm. Following purification by MPLC and semipreparative HPLC, the TSG concentration increased 3.75-fold, reaching 85,387.5 ± 192 ppm. LC–MS and ^1^H NMR analyses confirmed that TSG was the major constituent of the PME. Moreover, raw PM contained substantially higher TSG levels than processed materials, consistent with previous reports that identified TSG as the primary bioactive component responsible for promoting hair growth [31,32].

Negri-Cesi et al. [33] demonstrated that LNCaP cells express 5α-reductase, providing an effective model for investigating enzyme inhibition. In the present study, PME (1 µg/mL) inhibited 5α-reductase activity by 92.7%, exceeding the inhibitory efficacy of finasteride (77.2%) at the same concentration. In humans, DHT exhibits approximately fivefold higher affinity for androgen receptors in dermal papilla cells than TES [8]. Excessive 5α-reductase activity promotes the conversion of TES to DHT, leading to androgen receptor activation, follicular miniaturization, premature catagen entry, apoptosis, and ultimately AGA [34].

In vivo, PME significantly improved hair regrowth in TES-pretreated C57BL/6 mice, restoring the rate of follicular development to that observed in the control group. Histological examination revealed that PME-treated and minoxidil-treated mice developed mature, anagen-phase hair follicles comparable to those of the controls. In contrast, follicles in the TES group appeared shrunken, atrophic, and reduced in number, hallmarks of AGA [35,36]. The achievement of 92.7% inhibition of 5α-reductase activity by 1 μg/mL PME suggests that this formulation prevents DHT-mediated follicular degeneration by suppressing 5α-reductase activity, thereby maintaining normal follicular morphology and density. Consistent with this mechanism, both PME and minoxidil attenuated TES-induced suppression of new hair growth, though PME additionally targeted the enzymatic pathway responsible for DHT synthesis.

**Figure 8:**
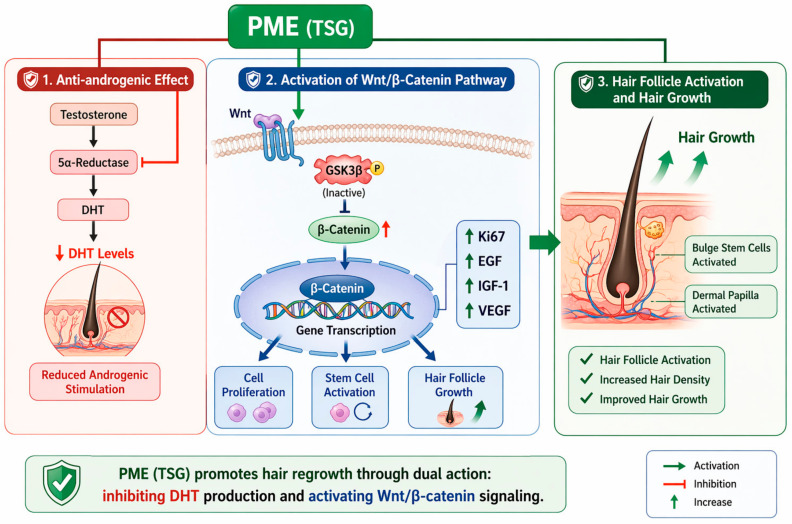
Schematic illustration depicting the proposed mechanism by which PME promotes hair regeneration in C57BL/6 mice with TES-induced androgenetic alopecia through activation of the Wnt/β-catenin signaling pathway via GSK3β inactivation and subsequent β-catenin accumulation.

PME also significantly enhanced 1.98-fold Ki67 expression, a marker of cellular proliferation, in follicular tissues. The hair growth cycle is tightly regulated by cytokines and growth factors such as IGF-1, which promotes the transition from telogen to anagen and prolongs the active growth phase [37,38]. PME treatment increased 1.21-fold IGF-1 expression, suggesting stimulation of follicular proliferation and anagen maintenance. Moreover, the mechanism by which PME promotes hair follicle regeneration is consistent with the Wnt/β-catenin signaling pathway. PME enhanced the phosphorylation of GSK3β at the Ser9 residue by 1.99-fold and increased β-catenin levels by 1.15-fold compared with TES-treated controls. The inactivation of GSK3β through Ser9 phosphorylation promotes the dissociation of β-catenin from the Axin/APC/GSK3β destruction complex, resulting in its cytoplasmic accumulation and subsequent nuclear translocation to activate transcription of hair growth-related genes. Biologically, activation of the GSK3β/β-catenin axis is critically involved in regulating the hair follicle growth cycle, particularly in triggering the transition from telogen to anagen and sustaining anagen maintenance. The observed upregulation of β-catenin is therefore indicative of enhanced follicular stem cell activation, dermal papilla signaling, and keratinocyte proliferation, all of which are essential for hair shaft elongation and follicular regeneration. Unlike classical Wnt/β-catenin pathway activators that directly stimulate Wnt ligand–receptor interactions or inhibit GSK3β activity through pharmacological blockade, PME appears to modulate this pathway indirectly by enhancing inhibitory phosphorylation of GSK3β, thereby providing a more physiologically regulated activation of β-catenin signaling. This mode of action may reduce the risk of aberrant β-catenin overactivation, which has been associated with follicular dysplasia and tumorigenesis, suggesting that PME-mediated Wnt/β-catenin activation is both effective and biologically balanced in promoting hair follicle growth. As illustrated in Figure 8, PME likely promoted GSK3β phosphorylation and β-catenin activation, thereby triggering Wnt/β-catenin pathway signaling [39,40]. This pathway activation is known to initiate the anagen phase and enhance hair shaft elongation [41].

In this study, PME exhibited a dual regulatory mechanism, combining potent 5α-reductase inhibition (92.7% at 1 μg/mL) with activation of the Wnt/β-catenin signaling pathway via inhibitory phosphorylation of GSK3β and increased β-catenin accumulation. Unlike finasteride, which lacks direct pro-regenerative signaling, and minoxidil, whose effects on Wnt/β-catenin activation are indirect, PME simultaneously suppresses androgen-driven follicular degeneration and promotes follicular regeneration, highlighting its potential as a balanced, multi-target hair growth-promoting strategy. Zhong et al. [42] explored the molecular mechanism of PM in treating androgenic alopecia based on the methods of bioinformatics and molecular docking. Anthraquinones and stilbenes are considered key components in PME’s nourishing effect on hair during AGA treatment [25]. Although TSG appears to be a key bioactive constituent responsible for the hair growth-promoting effects of PME, the present findings suggest that PME may exert greater biological efficacy than TSG alone. This enhanced activity is likely attributable to the presence of additional phytochemicals within PME that may act synergistically with TSG to modulate multiple signaling pathways involved in hair follicle regulation. Such compounds may contribute to improving the follicular microenvironment through antioxidant and anti-inflammatory effects, stabilizing Wnt/β-catenin signaling, or enhancing the bioavailability and cellular responsiveness to TSG [43,44]. Therefore, rather than acting as a single-agent effector, PME may promote hair growth through a coordinated, multi-component mechanism that more closely reflects the complexity of hair follicle biology.

Although the present study experimentally demonstrated the anti-androgenetic alopecia activity of TSG-enriched PME, further computational analyses, including network pharmacology, molecular docking, and molecular dynamics simulations, would provide additional insights into the interactions between major phytochemicals and therapeutic targets such as 5α-reductase, GSK3β, and β-catenin. These approaches may further elucidate the multi-target pharmacological mechanisms underlying the biological activity of PME and will be considered in future studies. In addition to male androgenetic alopecia, female-pattern hair loss (FPHL) has become a growing research focus [45,46]. Whether the underlying molecular mechanisms of FPHL mirror those of male alopecia remains unclear. Future investigations exploring the therapeutic potential of PME for FPHL and other forms of hair loss could provide valuable insights into its broader clinical applicability.

## 4. Materials and Methods

### 4.1. Preparation of PME

Dried roots of *P*. *multiflorum* Thunb (Purchased from a Taiwanese Chinese medicine shop, originating from Xi’an, China) were pulverized and sieved through a 100-mesh screen. *P. multiflorum* powder (2.0 g) was mixed with the designated volume of aqueous ethanol according to the selected solvent-to-solid ratio. Extraction was carried out using a laboratory microwave extraction system (MAS-II Plus, Sineo, Shanghai, China). During optimization, one extraction parameter (ethanol concentration, extraction time, extraction temperature, solvent-to-solid ratio, or microwave power) was varied while the remaining parameters were kept constant. Unless otherwise specified, the extraction conditions were 90% ethanol, 70 °C, 20 min, a solvent-to-solid ratio of 40 mL/g, and microwave power of 600 W. The optimized MAE conditions were subsequently compared with conventional reflux extraction, in which 2.0 g of *P. multiflorum* powder was extracted under different ethanol concentrations, extraction times, or solvent-to-solid ratios at 70 °C. The extract was then filtered and concentrated under reduced pressure prior to analysis. The content of TSG in each extract solution was quantified by high-performance liquid chromatography (HPLC; YL9110 Quaternary Pump, YL9131 Column Compartment, Autochro Data Module, YOUNG LIN, Seoul, Republic of Korea) at 280 nm and 35 °C. Separation was performed on a C18 column (Luna C18, 4.6 × 250 mm, 0.5 μm; Thermo Scientific, Middleton, WI, USA) using a gradient elution of water/acetonitrile (40:60 to 20:80, *v*/*v*) acidified with 0.1% formic acid.

The crude extract solution obtained by MAE was further purified using medium-pressure liquid chromatography (MPLC; C18, Cheetah MP200, Agela Technologies, Malvern, USA), followed by semipreparative high-performance liquid chromatography (HPLC; Luster C18, 10 × 250 mm, 5 μm; Dikmatech, Malvern, USA). Finally, the HPLC-purified PME was then freeze-dried into powder. All extractions and purifications were performed in accordance with the manufacturer’s protocols.

### 4.2. Extract Characterization

The chemical composition of HPLC-purified PME was identified using liquid chromatography–mass spectrometry (LC–MS; Orbitrap QE Plus, Thermo Scientific, Waltham, MA, USA) and proton nuclear magnetic resonance (^1^H-NMR; 300 MHz, Sedona, USA). All identification analyses were performed by the Precious Instruments Center of National Taiwan University.

### 4.3. Cell Culture and 5α-Reductase Extraction

LNCaP human prostate carcinoma cells (BCRC 60088) were obtained from the Bioresource Collection and Research Center (Hsinchu, Taiwan). Cells were cultured as monolayers in RPMI-1640 medium supplemented with 10% fetal bovine serum, 10 mM 4-(2-hydroxyethyl)-1-piperazineethanesulfonic acid (HEPES), 1.0 mM sodium pyruvate, and 1% penicillin–streptomycin. Cultures were maintained at 37 °C in a humidified incubator containing 5% CO_2_, with the medium replaced every 2–3 days. LNCaP cells, known to express high levels of 5α-reductase, were harvested at 80–90% confluency using 400 μL of trypsin and washed twice with phosphate-buffered saline (PBS). The cells were lysed with 200 μL of RIPA buffer, vortexed, and incubated on ice for 20 min. The lysate was centrifuged at 12,000 rpm for 20 min at 4 °C, and the supernatant containing 5α-reductase protein was collected for further analysis.

### 4.4. Quantification and Inhibition of 5α-Reductase Activity

Protein concentration was determined using an enzyme-linked immunosorbent BCA assay (Cloud-Clone Corp., Houston, TX, USA), following the manufacturer’s instructions. A total of 200 μL of working solution was added to the standard and protein samples and incubated at 37 °C for 30 min. Absorbance was measured at 450 nm, and protein concentration was calculated from the standard curve.

The inhibitory activity of PME on 5α-reductase was evaluated following a modified method from Chaiyana et al. [47], by measuring residual TES. The reaction volume was 550 μL (Table 1) and consisted of TES (0.02 mg/mL, 200 μL), PME (50 μL; various concentrations) or finasteride (Fin, 1 μg/mL), NADPH (0.835 mg/mL, 200 μL), and Tris buffer (50 μL). The mixture was preincubated in a 50 °C water bath for 20 min, after which 5α-reductase solution (50 μL) was added and the reaction was maintained at 37 °C for 60 min.

The reaction was terminated by adding 2 mL of ethyl acetate, followed by centrifugation at 5000 rpm for 10 min. The supernatant (1.9 mL) was evaporated and redissolved in 100 μL of acetonitrile. TES content was analyzed via HPLC, and enzyme inhibition (%) was calculated using Equation (1), where C0 and C60 are the concentrations of TES after 0 and 60 min of reaction, respectively, and Sample is PME or Fin.

(1) $$\mathrm{Inhibition}\;(\%)=\frac{C_{\mathrm{extract}}}{C_{\mathrm{control}}}\times 100$$

where $C_{\mathrm{extract}}={\mathrm{TES}}_{\mathrm{sample}}-{\mathrm{TES}}_{C60}$ and $C_{\mathrm{control}}={\mathrm{TES}}_{C0}-{\mathrm{TES}}_{C60}$.

### 4.5. In Vivo Hair Growth Experiment

The effect of PME on hair regrowth was evaluated in TES-treated C57BL/6 mice (7 weeks old), following Kamei et al. [48] with slight modifications. Mice were obtained from BioLasco Taiwan and acclimatized for one week before experimentation. All animals were housed under controlled temperature and humidity with a 12 h light/dark cycle and had free access to food and water.

All experimental procedures were approved by the Institutional Animal Care and Use Committee (IACUC) of Fu Jen Catholic University (Approval No. A10768, Taipei, Taiwan), and this study was reported in accordance with the ARRIVE 2.0 guidelines. Mice were anesthetized using isoflurane (4% induction, 1% maintenance). To synchronize hair growth, dorsal hair was shaved, and residual hair was removed using depilatory cream.

8 mice were randomly divided into four groups (n = 2 per group): control (PBS, 100 μL), TES (0.02 mg/mL, 100 μL), TES (0.02 mg/mL, 100 μL) + minoxidil (2%, 100 μL), and TES (0.02 mg/mL, 100 μL) + PME (20 mg/mL, 100 μL). The solution was applied topically to the back of each mouse daily, while the test group received TES once. After 30 min, minoxidil or PME was applied, and the treatment was continued for 28 days. Hair growth was scored visually on a 0–5 scale: 0 (0%), 1 (0–20%), 2 (20–40%), 3 (40–60%), 4 (60–80%), and 5 (80–100%) regrowth [49]. On day 28, mice were euthanized with CO_2_, and dorsal skin samples were collected for hematoxylin–eosin (H&E) staining and Western blot analysis.

### 4.6. Western Blot Analysis

Skin tissues were ground in liquid nitrogen and lysed in RIPA buffer (1:10, g/mL). The lysates were centrifuged at 12,000 rpm for 20 min at 4 °C, and the supernatants were collected. Protein concentration was determined by BCA assay at 595 nm.

Equal amounts of protein were separated on 12% SDS–polyacrylamide gels and transferred onto polyvinylidene difluoride (PVDF) membranes (Millipore, Sedona, AZ, USA). Membranes were blocked and incubated overnight at 4 °C with primary antibodies (1:1000; Affinity Biosciences, Cincinnati, OH, USA): β-actin, GSK3β (Ser9; AF2016), β-catenin (AF6266), Ki67 (AF0198), epidermal growth factor (EGF; AF5148), insulin-like growth factor-1 (IGF-1; AF3123), and vascular endothelial growth factor (VEGF; AF5131). After washing, membranes were incubated with horseradish peroxidase (HRP)-conjugated goat anti-rabbit IgG secondary antibodies (1:80,000; Sigma-Aldrich, Burlington, MA, USA) for 1 h at room temperature. Protein bands were visualized using Western Lightning ECL Pro substrate (PerkinElmer, Waltham, MA, USA) and imaged via chemiluminescence. Band intensities were quantified using ImageJ software (Version 1.54k, NIH, Bethesda, MD, USA), with β-actin serving as the internal control.

### 4.7. AI Usage

We used ChatGPT (version GPT-5.5, OpenAI, San Francisco, CA, USA) to create AG and Figure 8 from the experimental data and results, to convey the research content to the readers more clearly.

### 4.8. Statistical Analysis

All experiments were conducted in triplicate. Data are expressed as the mean ± standard deviation (SD). Statistical analyses were performed using IBM SPSS Statistics (version 23). Differences among groups were analyzed using one-way ANOVA followed by Dunnett’s test. A *p*-value < 0.05 was considered statistically significant.

## 5. Conclusions

This study successfully established an optimized microwave-assisted extraction and chromatographic purification procedure for obtaining TSG-enriched PME, and confirmed its chemical identity by LC-MS and ^1^H NMR spectroscopy. The purified PME exhibited potent 5α-reductase inhibitory activity, thereby preventing the conversion of TES to DHT. In vivo, PME enriched with TSG promoted hair growth by increasing the size and number of hair follicles in TES-treated mice, primarily through upregulation of β-catenin expression via activation of the Wnt/β-catenin signaling pathway. These findings suggest that TSG is a key bioactive constituent of PME and may serve as a promising therapeutic agent for the treatment of androgenetic alopecia and related hair loss disorders.

## Figures and Tables

**Table 1:** 5α-Reductase activity inhibition: reaction composition and time in each group.

	C60	C0	Sample
Testosterone (0.02 mg/mL)	200 μL	200 μL	200 μL
tris buffer	100 μL	350 μL	50 μL
NADPH (0.835 mg/mL)	200 μL	-	200 μL
5α-reductase (1.6 mg/mL)	50 μL	-	50 μL
PME (0.05~1 μg/mL) or Fin (1 μg/mL)	-	-	50 μL
Total	550 μL	550 μL	550 μL
Time (min)	60	0	60

## Data Availability

The original contributions presented in this study are included in the article. Further inquiries can be directed to the corresponding author.

## References

[1] Han S.H., Byun J.W., Lee W.S., Kang H., Kye Y.C., Kim K.H., Kim D.W., Kim M.B., Kim S.J., Kim H.O. (2012). Quality of life assessment in male patients with androgenetic alopecia: Result of a prospective, multicenter study. Ann. Dermatol..

[2] Filaire E., Dreux A., Boutot C., Ranouille E., Berthon J.Y. (2020). Characteristics of healthy and androgenetic alopecia scalp microbiome: Effect of *Lindera strychnifolia* roots extract as a natural solution for its modulation. Int. J. Cosmet. Sci..

[3] Kranz D. (2011). Young men’s coping with androgenetic alopecia: Acceptance counts when hair gets thinner. Body Image.

[4] Chen X., Liu B., Li Y., Han L., Tang X., Deng W., Lai W., Wan M. (2019). Dihydrotestosterone regulates hair growth through the Wnt/β-Catenin Pathway in C57BL/6 mice and in vitro organ culture. Front. Pharmacol..

[5] Chenu C., Marine A., Frederic B., Elodie C., Uihot A.L., Toutain C., Isabelle R.L., Patricia V., Gadeau A.P., Danie H. (2017). Testosterone prevents cutaneous ischemia and necrosis in males through complementary estrogenic and androgenic actions. Arter. Thromb. Vasc. Biol..

[6] Culley C., Roger R. (2003). The role of dihydrotestosterone in benign prostatic hyperplasia. Urology.

[7] Keith D.K. (2002). Androgens and alopecia. Mol. Cell. Endocrinol..

[8] Ralph M.T. (2002). Molecular mechanisms of androgenetic alopecia. Exp. Gerontol..

[9] Melissa C.J., Pharm D. (2008). Treatment options for androgenetic alopecia. US Pharm..

[10] Santos A.C., Miguel P.S., Catarina G., Diana C.P., Diana P., Irina C., Ribeiro A.J., Francisco V. (2020). Topical minoxidil-loaded nanotechnology strategies for alopecia. Cosmetic.

[11] Fang C., Yang H., Hui J., Fan D., Deng J. (2024). Natural bioactive compounds as potential sources for alleviation and treatment of androgenetic alopecia: A research advance. eFood.

[12] Elnady R.E., Abdon M.S., Shaheen H.R., Eladawy R.M., Azar Y.O., Al Raish S.M. (2025). The future of alopecia treatment: Plant extracts, nanocarriers, and 3D bioprinting in focus. Pharmaceutics.

[13] Al Raish S.M., Alsheriafi H.N., Alkuwaiti A.A., Safi S.K., Safi A.S. (2026). Insights into knowledge, attitudes, and practices of medicinal plant use in the United Arab Emirates: A cross-sectional study. Front. Nutr..

[14] Zhao Y.K., Guo R.X., Li R.S., Shi W., Gong H.Y., Ma R.R., Gao H., Li Z., Hu K.J., Bai Z.F. (2025). Investigation on the therapeutic effect of *Polygonum multiflorum* Thunb. in chronic stress-induced hair loss in mice coupled with metabolomics and proteomics. World J. Tradit. Chin. Med..

[15] Almasri R.S., Bedir A.S., Al Raish S.M. (2025). Comprehensive Ethnopharmacological Analysis of Medicinal Plants in the UAE: Lawsonia inermis, Nigella sativa, Ziziphus spina-christi, Allium cepa, Allium sativum, Cymbopogon schoenanthus, Matricaria aurea, Phoenix dactylifera, Portulaca oleracea, Reichardia tingitana, Salvadora persica, Solanum lycopersicum, Trigonella foenum-graecum, Withania somnifera, and Ziziphus lotus. Nutrients.

[16] Han S.Y., Lee K.Y., Kim Y.K. (2018). *Poligoni Multiflori* Radix enhances osteoblast formation and reduces osteoclast differentiation. Int. J. Mol. Med..

[17] Yang J.B., Li W.F., Liu Y., Wang Q., Cheng X.L., Wei F., Wang A.G., Jin H.T., Mas C. (2018). Acute toxicity screening of different extractions, components and constituents of *Polygonum multiflorum* Thunb. on zebrafish (*Danio rerio*) embryos in vivo. Biomed. Pharmacother..

[18] Kang Y., Lee K., Choi J., Komakech R. (2018). Maximizing seedling and root tuber production in *Polygonum multiflorum* for use in ethnomedicine. S. Afr. J. Bot..

[19] Liu Y., Sun Y., Huang G. (2018). Preparation and antioxidant activities of important traditional plant polysaccharides. Int. J. Biol. Macromol..

[20] Yao W., Fan W., Huang C., Zhong H., Chen X., Zhang W. (2013). Proteomic analysis for anti-atherosclerotic effect of tetrahydroxystilbene glucoside in rats. Biomed. Pharmacother..

[21] Guo Y., Fan W., Cao S., Xie Y., Hong J., Zhou H., Wan H., Jin B. (2020). 2,3,5,4′-Tetrahydroxystilbene-2-O-β-D-Glucoside modulated human umbilical vein endothelial cells injury under oxidative stress. Korean J. Physiol. Pharmacol..

[22] Wang X., Zhao L., Han T., Chen S., Wang J. (2008). Protective effects of 2,3,5,4′-tetrahydroxystilbene-2-O-beta-d-glucoside, an active component of *Polygonum multiflorum* Thunb, on experimental colitis in mice. Eur. J. Pharmacol..

[23] Chen L., Duan H., Xie F., Gao Z., Wu X., Chen F., Wu W. (2018). Tetrahydroxystilbene glucoside effectively prevents apoptosis induced hair loss. BioMed Res. Int..

[24] Qian J., Feng C., Wu Z., Yang Y., Gao X., Zhu L., Liu Y., Gao Y. (2024). Phytochemistry, pharmacology, toxicology and detoxification of *Polygonum multiflorum* Thunb.: A comprehensive review. Front. Pharmacol..

[25] Han B., Xiao M., Xin T., Hu H., Liu Q., Xu B. (2025). Research progress on the application of *Pleuropterus multiflorus* in the treatment of androgenetic alopecia. J. Holist. Integr. Pharm..

[26] Huang T.Y., Huang M.Y., Tsai C.K., Su W.T. (2021). Phosphorylation of levan by microwave-assisted synthesis enhanced anticancerability. J. Biosci. Bioeng..

[27] Xiao W., Han L., Shi B. (2008). Microwave-assisted extraction of flavonoids from *Radix astragali*. Sep. Purif. Technol..

[28] Kim D., Park S. (2020). Pharmacological therapeutics in androgenetic alopecia. J. Korean Med. Assoc..

[29] Varghese F., Bukhari A.B., Malhotra R., De A. (2014). IHC Profiler: An open source plugin for the quantitative evaluation and automated scoring of immunohistochemistry images of human tissue samples. PLoS ONE.

[30] Zhang L., Tao N., Chen Y., Xu J., Chen Z., Zhang G., Gao J. (2026). Tetrahydroxy stilbene glucoside promotes hair regeneration by inducing Th22 cell differentiation. J. Ethnopharmacol..

[31] Park H.J., Zhang N., Park D.K. (2011). Topical application of *Polygonum multiflorum* extract induces hair growth of resting hair follicles through upregulating Shh and β-catenin expression in C57BL/6 mice. J. Ethnopharmacol..

[32] Shin J.Y., Choi Y.H., Kim J., Park S.Y., Nam Y.J., Lee S.Y., Jeon J.H., Jin M.H., Lee S. (2020). *Polygonum multiflorum* extract support hair growth by elongating anagen phase and abrogating the effect of androgen in cultured human dermal papilla cells. BMC Complement. Med. Ther..

[33] Negri-Cesi P., Poletti A., Colciago A., Magni P., Martini P., Motta M. (1998). Presence of 5α-Reductase isozymes and aromatase in human prostate cancer cells and in benign prostate hyperplastic tissue. Prostate.

[34] Herman A., Herman A.P. (2016). Mechanism of action of herbs and their active constituents used in hair loss treatment. Fitoterapia.

[35] Truong V.L., Bak M.J., Lee C., Jun M., Jeong W.S. (2017). Hair regenerative mechanisms of red ginseng oil and its major components in the testosterone-induced delay of anagen entry in C57BL/6 mice. Molecules.

[36] Lin Y., Liu C., Zhan X., Wang B., Li K., Li J. (2020). Jagged1 and epidermal growth factor promoted androgen- suppressed mouse hair growth in vitro and in vivo. Front. Pharmacol..

[37] Lee N.E., Park S.D., Hwang H., Choi S.H., Lee R.M., Nam S.M., Choi J.H., Rhim H., Cho I.H., Kim H.C. (2020). Effects of a gintonin-enriched fraction on hair growth: An in vitro and in vivo study. J. Ginseng Res..

[38] Woo Y.M., Kim O.J., Jo E.S., Jo M.Y., Ahn M.Y., Lee Y.H., Li C.R., Lee S.H., Choi J.S., Ha J.M. (2019). The effect of *Lactobacillus plantarum* hydrolysates promoting VEGF production on vascular growth and hair growth of C57BL/6 mice. J. Anal. Sci. Technol..

[39] Tata P.R., Michael K. (2010). An Updated overview on Wnt signaling pathways. Circ. Res..

[40] Ahn J.H., Park Y.E., Kim B., Park C.W., Sim T.H., Lee T.K., Lee J.C., Park J.H., Kim J.D., Lee H.S. (2020). Hair growth is promoted in mouse dorsal skin by a mixture of *Platycladus orientalis* (L.) Franco Leaf extract and alpha-terpineol by increasing growth factors and wnt3/β-Catenin. Nat. Prod. Commun..

[41] Kang J.I., Choi Y.K., Koh Y.S., Hyun J.W., Kang J.H., Lee K.S., Lee C.M., Yoo E.S., Kang H.K. (2020). Vanillic acid stimulates anagen signaling via the PI3K/Akt/β-Catenin pathway in dermal papilla cells. Biomol. Ther..

[42] Zhong X., Jing C. (2025). Exploring the molecular mechanism of *Polygonum multiflorum* in treating androgenic alopecia based on the methods of bioinformatics and molecular docking. Medicine.

[43] Zhu C., Li J., Tang W., Li Y., Lin C., Peng D., Yang C. (2025). 2,3,5,4′-Tetrahydroxystilbene-2-O-β-D-glucoside (TSG) from *Polygonum multiflorum* Thunb.: A Systematic Review on Anti-Aging. Int. J. Mol. Sci..

[44] Li Y., Han M., Lin P., He Y., Yu J., Zhao R. (2015). Hair Growth Promotion Activity and Its Mechanism of *Polygonum multiflorum*. Evid. Based Complement. Altern. Med..

[45] Ho C.Y., Chen J.Y.F., Hsu W.L., Yu S., Chen W.C., Chiu S.H., Yang H.R., Lin S.Y., Wu C.Y. (2023). Female pattern hair loss: An overview with focus on the genetics. Genes.

[46] Wang H.J., Yeh J.W., Chang Y.F., Wu J.S., Yang C.C. (2022). Comorbid laboratory abnormalities in female pattern hair loss patients. Dermatol. Sin..

[47] Chaiyana W., Punyoyai C., Somwongin S., Leelapornpisid P., Ingkaninan K., Waranuch N., Srivilai J., Thitipramote N., Wisuitiprot W., Schuster R. (2017). Inhibition of 5α-reductase, IL-6 secretion, and oxidation process of *Equisetum debile* Roxb. ex. vaucher extract as functional food and nutraceuticals ingredients. Nutrients.

[48] Kamei H., Noguchi K., Matsuda H., Murata K. (2018). Screening of *Euphorbiaceae* plant extracts for anti-5α-reductase. Biol. Pharm. Bull..

[49] Chang W.C., Chen K.Y., Cheng S.Y., Hsu C.K., Yang C.C. (2023). Mapping the hair density, thickness, and volume in normal and androgenetic alopecia subjects with the digital microscope. JEADV Clin. Pract..

